# Combined use of GORE TAG^®^ and Gore Exculder^®^ endografts for treatment of abdominal aortic aneurysm with severe angulation

**DOI:** 10.1590/S1679-45082014RC2788

**Published:** 2014

**Authors:** Mariana Krutman, Cynthia de Almeida Mendes, Flavio Henrique Duarte, Kenji Nishinari, Nelson Wolosker

**Affiliations:** 1 Hospital Israelita Albert Einstein, São Paulo, SP, Brazil.; 2 Hospital das Clínicas, Faculdade de Medicina, Universidade de São Paulo, São Paulo, SP, Brazil.; 3 Hospital Antônio Cândido de Camargo, São Paulo, SP, Brazil.

**Keywords:** Aortic aneurysm, abdominal/therapy, Aortic aneurysm, abdominal/surgery, Endovascular procedures/methods, Case reports

## Abstract

The advances in endovascular surgery for treatment of aortic aneurysms have allowed a greater number of patients, who were previously considered unsuitable for the approach, to benefit from this therapeutic modality. Despite the current availability of highly comfortable endografts, cases with unfavorable anatomy remain a challenge for surgeons. We report a case with difficult anatomy that was successfully managed using an unconventional endovascular technique.

## INTRODUCTION

Population aging has as a major consequence the great number of elderly candidates for abdominal aortic aneurysm (AAA) repair surgery^.^^(1)^ Technological advances enabled the development of modern endografts with high capacity of adaptation to unfavorable anatomies, and allowed endovascular treatment to become an option for patients in an advanced stage of the disease with large, angulated and tortuous aneurysms^.^^(2)^

Despite the current availability of highly conformable endografts some cases with unfavorable anatomy do not permit endovascular repair, which lead surgeons to seek for non-conventional endovascular alternatives for AAA treatment.

We report a case of an 89-years-old woman with a large, extensive (18.5cm in length between renal arteries and aortic bifurcation) and angulated infrarenal AAA, successfully treated with an unconventional endovascular technique.

## CASE REPORT

**Figure 1:**
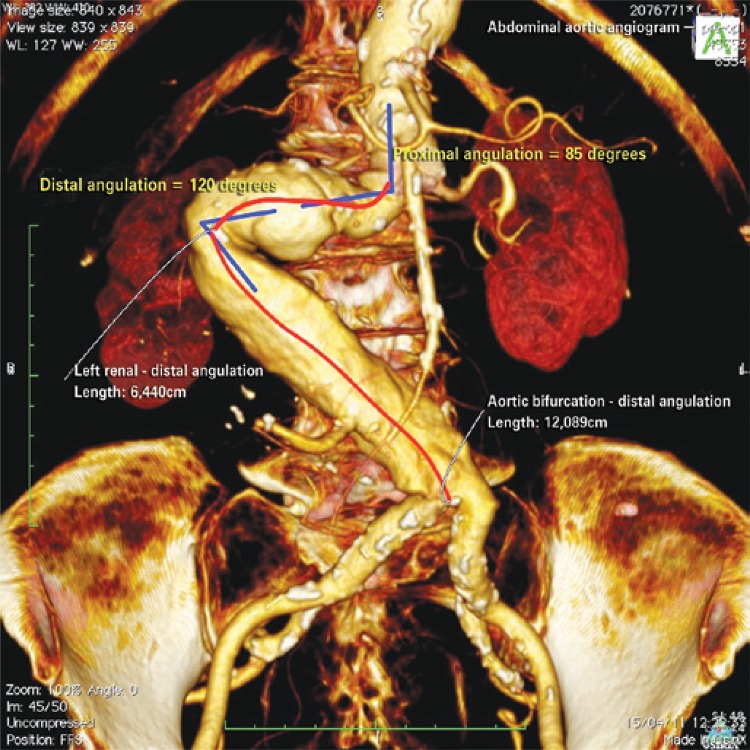
Preoperative angiotomography showing aneurysm length and tortuosity

An 89-year-old woman without comorbidities was submitted to a routine abdominal ultrasonography exam that showed an infrarenal AAA. Because the patient’s renal function was preserved, an aortic angiotomography was done to complete the diagnosis. The angiotomography showed an aneurysm with a 5.6cm diameter, an extremely long and tortuous body, and two major angulations. The proximal angulation at the renal level measured 85° and the distal angulation measured 120° with respect to the longitudinal axis of the aorta, 6cm below the left renal. The total length between the lower left renal and the aortic bifurcation was 18.5cm, being 6.4cm from the neck to the distal angulation and 12.1cm from the distal angulation to the aortic bifurcation (Figure 1).

The patient underwent endovascular aneurysm repair under general anesthesia using a non-conventional surgical approach. We chose to initially deploy the GORE TAG^®^ 26x10 thoracic endograft in order to treat the proximal neck, below the renal arteries. The choice of the thoracic endograft was intended to follow the aortic curvature, allow the “creation” of a new, more rectified proximal neck, and facilitate the fixation of abdominal endografts inside. We used a Gore Exculder^®^ 31x14x17 abdominal graft that was released inside the thoracic endoprothesis with good fixation and 3 a cm overlap. A contralateral branch GORE^®^ 16x12 was used to cover the left common iliac artery.

The final intraoperative control arteriography showed total exclusion of aneurysm sac and absence of endoleaks.

**Figure 2:**
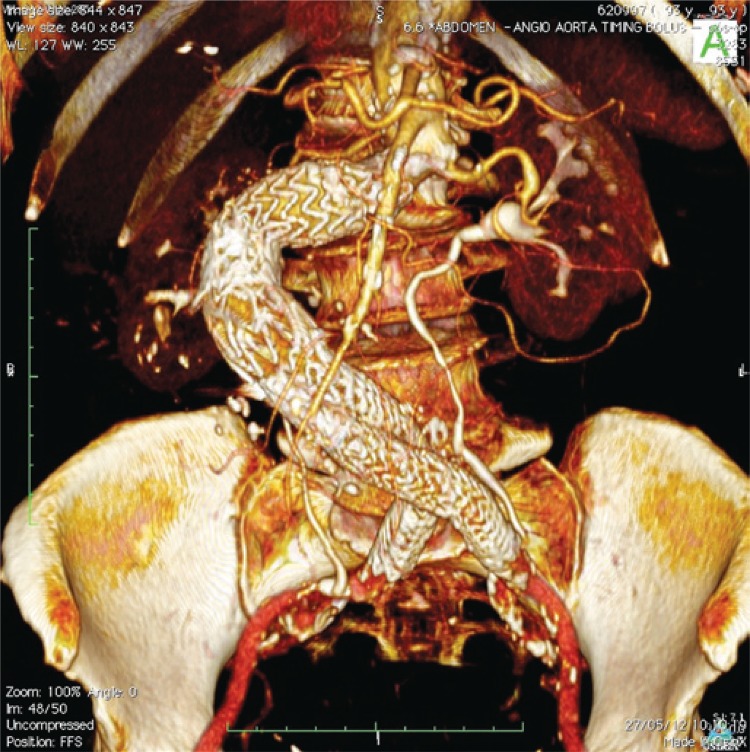
Tomographic control 1 year after surgery showing good positioning of the endografts without endoleaks

The patient had an uneventful recovery after surgery and was discharged 3 days after the procedure. A control angiotomography conducted 1 year after the surgery showed reduction of the aneurysmal sac without endoleaks or any other signs of surgical complications (Figure 2).

## DISCUSSION

Similar to peripheral arterial occlusive disease, the endovascular AAA repair has been replacing the conventional surgery for its low perioperative morbimortality rate, mainly in individuals aged ≥80 years.^(2)^ The development of new endoprosthesis, with greater flexibility and better fixation to the aortic wall, has been enlarging the possibility to offer a less invasive treatment option for patients who were previously considered unfit for endovascular repair.

Surgical indication in our patient was based on the size of aneurysm and her good clinical conditions, not requiring conservative measures. Benefits of an endovascular technique were undoubtful in our patient especially because of her advanced age; however, the unfavorable anatomical conditions prevented this approach with the standard commercially available endografts.

General anatomic criteria that hinder endovascular approach include short and dilated neck; presence of neck thrombus; accentuated angulations involving the neck, body and aortoiliac axis; and iliac-femoral atherosclerotic disease. Our patient had two limiting anatomical characteristics: extremely extended length of body/neck and accentuated angulations.

The release of a conventional abdominal endograft in that aneurysm would result in positioning of the main body bifurcation just above distal angulation, making contralateral catheterization extremely difficult. Even if this catheterization was made possible, it would pose a considerable risk of branch occlusion due to the possibility of branch folding at the angulation site.

The use of thoracic endografts to facilitate proximal fixation in abdominal aneurysms was already described in the literature.^(3-5)^ Variations of surgical technique have been described regarding the deployment of thoracic endografts before or after implantation of the main abdominal body, and both techniques presented good results. However, the specific aneurysm anatomy observed in our case, does not resemble the characteristics observed in previous reports and the use of GORE^®^ endografts has never been described for such specific purpose.

Our strategy enabled the use of a thoracic endograft in abdominal region, respecting local anatomical conditions and ensuring a safe, effective and minimally invasive treatment of an aneurysm with potential for rupture.
