# Supplementary material for: Combined use of GORE TAG® and Gore Exculder® endografts for treatment of abdominal aortic aneurysm with severe angulation
Source: Einstein (Sao Paulo). 2014 Oct-Dec;12(4):499–501. doi: 10.1590/S1679-45082014RC2788 (PMC4879919; doi:10.1590/S1679-45082014RC2788)
Supplement: Translation

# Translation: Uso combinado de endoprótese GORE TAG^®^ e Gore Exculder^®^ para tratamento de aneurisma de aorta abdominal com angulação extrema

**Authors:** Mariana Krutman, Cynthia de Almeida Mendes, Flavio Henrique Duarte, Kenji Nishinari, Nelson Wolosker

**Affiliations:** 1 Hospital Israelita Albert Einstein, São Paulo, SP, Brasil.; 2 Hospital das Clínicas, Faculdade de Medicina, Universidade de São Paulo, São Paulo, SP, Brasil.; 3 Hospital Antônio Cândido de Camargo, São Paulo, SP, Brasil.

**Keywords:** Aneurisma de aorta abdominal/terapia, Aneurisma de aorta abdominal/cirurgia, Procedimentos endovasculares/métodos, Relatos de casos

## Abstract

O avanço tecnológico da cirurgia endovascular no tratamento de aneurismas de aorta vem permitindo que uma maior quantidade de pacientes, antes considerados inaptos para essa abordagem, beneficie-se dessa modalidade terapêutica. Apesar da atual disponibilidade de endopróteses com alta conformabilidade, casos com anatomia desfavorável permanecem um desafio para os cirurgiões. Descrevemos um caso anatomicamente desfavorável, resolvido com sucesso pela técnica endovascular, utilizando-se uma manobra não convencional.

## INTRODUÇÃO

O envelhecimento da população tem como consequência um maior número de idosos candidatos à correção cirúrgica de aneurismas da aorta abdominal (AAA).^(1)^ O avanço tecnológico permitiu o desenvolvimento de modernas endopróteses, com alta capacidade de adaptação a anatomias desfavoráveis, tornando o tratamento endovascular a opção de escolha nesses pacientes, que, frequentemente, apresentam-se em fase avançada da doença, com aneurisma grandes, angulados e tortuosos.^(2)^

Apesar da atual disponibilidade de endopróteses com alta conformabilidade, em alguns casos a anatomia é tão desfavorável que não permite a correção endovascular, fazendo com que o cirurgião busque alternativas não convencionais para sua resolução.

Apresentamos o caso de uma paciente de 89 anos, portadora de um AAA infrarrenal muito longo (extensão de 18,5cm entre as artérias renais e a bifurcação da aorta) e angulado, resolvido com sucesso pela técnica endovascular utilizando-se uma manobra não convencional.

## RELATO DE CASO

Paciente de 89 anos, gênero feminino, sem comorbidades, foi submetida a exame ultrassonográfico abdominal de rotina, que evidenciou achado de AAA infrarrenal. Como a função renal encontrava-se preservada, optou-se por realizar angiotomografia da aorta para complementação diagnóstica. O exame revelou aneurisma com diâmetro máximo de 5,6cm e corpo extremamente alongado e tortuoso, apresentando duas angulações principais, sendo a proximal de 85° no nível das renais, e a distal de 120° com o eixo longitudinal da aorta, 6cm abaixo da renal esquerda. O comprimento total entre a renal esquerda (mais baixa) e a bifurcação aórtica era de 18,5cm, sendo divida em 6,4cm até a angulação distal e mais 12,1cm até a bifurcação da aorta, conforme visualizado na figura 1.

**Figura 1:** Angiotomografia pré-operatória evidenciando comprimento e tortuosidade do aneurisma

A paciente foi submetida à correção endovascular do aneurisma, sob anestesia geral, utilizando uma tática operatória não convencional. Optamos pela liberação inicial de uma endoprótese torácica GORE TAG^®^ 26x10 visando ao tratamento do colo proximal, logo abaixo das artérias renais. A endoprótese torácica escolhida teve como finalidade acompanhar a curvatura da aorta, possibilitando a “criação” de um novo colo proximal mais retificado, facilitando a fixação da endoprótese abdominal em seu interior. Utilizamos uma prótese abdominal Gore Exculder^®^ 31x14x17, que foi liberada dentro da endoprótese torácica previamente implantada, com boa fixação e sobreposição de 3cm entre elas. Um ramo contralateral GORE^® ^16x12 foi utilizado para complementar a cobertura da artéria ilíaca comum esquerda.

A arteriografia intraoperatória final mostrou exclusão total do aneurisma e ausência de vazamentos.

A paciente evoluiu bem no pós-operatório, recebendo alta no 3^o^ dia após o procedimento, sem intercorrências. A angiotomografia de controle realizada 1 ano após a cirurgia evidenciou redução do diâmetro máximo do saco aneurismático sem sinais de vazamentos ou demais complicações cirúrgicas (Figura 2).

**Figura 2:** Controle tomográfico 1 ano após cirurgia, evidenciando bom posicionamento das endopróteses, sem extravasamentos

## DISCUSSÃO

Da mesma forma que na doença arterial obstrutiva periférica, a correção endovascular de AAA vem substituindo a cirurgia convencional, devido à sua menor morbimortalidade perioperatória, especialmente em indivíduos com idade superior a 80 anos.^(2)^ O desenvolvimento de novas endopróteses, mais flexíveis e com melhor fixação à parede da aorta, vem ampliando a possibilidade de oferecer um tratamento menos invasivo à pacientes antes considerados inapropriados para correção endovascular.

A indicação cirúrgica para a paciente em questão baseou-se no tamanho do aneurisma e nas boas condições clínicas da paciente, que não justificariam a opção por uma conduta conservadora. Não havia dúvida de que essa paciente se beneficiaria do tratamento endovascular em virtude de sua idade avançada, entretanto as condições anatômicas desfavoráveis inviabilizariam o método com o material padronizado atualmente disponível no mercado.

Critérios anatômicos gerais que dificultam a abordagem endovascular incluem colo curto e dilatado; presença de trombo mural junto ao colo; angulações acentuadas envolvendo o colo, corpo e eixo aorto-ilíaco e doença aterosclerótica ilíaco-fermoral. A paciente do caso apresentava duas características anatômicas limitantes: extensão extremamente alongada do corpo/colo e angulações acentuadas no trajeto do aneurisma.

A liberação de uma endoprótese abdominal convencional nesse aneurisma resultaria no posicionamento da bifurcação do corpo principal, logo acima da angulação distal, tornando a cateterização contralateral muito difícil. Mesmo que essa cateterização fosse factível, haveria risco considerável de oclusão dos ramos em virtude da eventual dobra dos mesmos no local da angulação.

A utilização de endopróteses torácicas, para facilitar a fixação proximal em aneurismas abdominais, já foi previamente descrita na literatura.^(3-5)^ Variações da técnica cirúrgica foram exploradas, com liberação da endoprótese torácica antes ou após o implante do corpo principal abdominal, ambas com bom resultados. Entretanto, a anatomia peculiar, observada nesse caso específico, não se equipara às características observadas nos relatos preexistentes e a utilização da endopróstese GORE^®^ nunca foi descrita para essa finalidade.

A estratégia adotada permitiu a utilização de uma prótese torácica em região abdominal, respeitando as condições anatômicas locais e garantido o tratamento seguro, efetivo e minimamente invasivo de uma aneurisma com potencial para rotura.
